# Increased Surface Roughness in Polydimethylsiloxane Films by Physical and Chemical Methods

**DOI:** 10.3390/polym9080331

**Published:** 2017-08-02

**Authors:** Jorge Nicolás Cabrera, Mariano M. Ruiz, Mirta Fascio, Norma D’Accorso, Rosica Mincheva, Philippe Dubois, Leonardo Lizarraga, R. Martín Negri

**Affiliations:** 1 Instituto de Química Física de Materiales, Ambiente y Energía (INQUIMAE), Ciudad Autónoma de Buenos Aires C1428EGA, Argentina; nicoo.cabrera@yahoo.com.ar (J.N.C.); marianoruiz@qi.fcen.uba.ar (M.M.R.); 2 Departamento de Química Inorgánica, Analítica y Química Física, Facultad de Ciencias Exactas y Naturales, Universidad de Buenos Aires (UBA), Ciudad Autónoma de Buenos Aires C1428EGA, Argentina; 3 Centro de Investigación en Hidratos de Carbono (CIHIDECAR-CONICET), Ciudad Autónoma de Buenos Aires C1428EGA, Argentina; mfascio@qo.fcen.uba.ar (M.F.); norma@qo.fcen.uba.ar (N.D.); 4 Departamento de Química Orgánica, Facultad de Ciencias Exactas y Naturales, UBA, Ciudad Autónoma de Buenos Aires C1428EGA, Argentina; 5 Center of Innovation and Research in Materials & Polymers (CIRMAP), University of Mons, 7000 Mons, Belgium; rosica.mincheva@umons.ac.be (R.M.); philippe.dubois@umons.ac.be (P.D.); 6 Centro de Investigaciones en Bionanociencias (CIBION-CONICET), Godoy Cruz 2390, 1st Floor, Ciudad Autónoma de Buenos Aires C1425FQD, Argentina

**Keywords:** composites, roughness, magnetism, bromine

## Abstract

Two methods, the first physical and the other chemical, were investigated to modify the surface roughness of polydimethylsiloxane (PDMS) films. The physical method consisted of dispersing multi-walled carbon nanotubes (MWCNTs) and magnetic cobalt ferrites (CoFe_2_O_4_) prior to thermal cross-linking, and curing the composite system in the presence of a uniform magnetic field **H**. The chemical method was based on exposing the films to bromine vapours and then UV-irradiating. The characterizing techniques included scanning electron microscopy (SEM), energy-dispersive spectroscopy (EDS), Fourier transform infrared (FTIR) spectroscopy, optical microscopy, atomic force microscopy (AFM) and magnetic force microscopy (MFM). The surface roughness was quantitatively analyzed by AFM. In the physical method, the random dispersion of MWCNTs (1% *w*/*w*) and magnetic nanoparticles (2% *w*/*w*) generated a roughness increase of about 200% (with respect to PDMS films without any treatment), but that change was 400% for films cured in the presence of **H** perpendicular to the surface. SEM, AFM and MFM showed that the magnetic particles always remained attached to the carbon nanotubes, and the effect on the roughness was interpreted as being due to a rupture of dispersion randomness and a possible induction of structuring in the direction of **H**. In the chemical method, the increase in roughness was even greater (1000%). Wells were generated with surface areas that were close to 100 μm^2^ and depths of up to 500 nm. The observations of AFM images and FTIR spectra were in agreement with the hypothesis of etching by Br radicals generated by UV on the polymer chains. Both methods induced important changes in the surface roughness (the chemical method generated the greatest changes due to the formation of surface wells), which are of great importance in superficial technological processes.

## 1. Introduction

The modification of polymer surfaces finds applications in many relevant areas, such as the development of super-hydrophobic membranes for oil–water separation [1,2], activation surfaces for microfluidics [3], the attachment of biomolecules for biosensors [4], self-replenishing coatings [5], and anti-bacterial and fouling release coatings [6,7,8]. Such modification to increase the surface roughness is a topic of permanent interest, as it may favor or disfavor the adsorption of macromolecules or the formation of biofilms on the polymer surface. In fact, the motivation for the present work is related to the interesting antifouling properties of polydimethylsiloxane (PDMS) surfaces and the possibilities to change these by modifying the roughness. The low surface energy of silicone materials makes them useful in the production of non-sticking and/or readily cleanable coatings in aqueous environments [9]. However, despite the inherently good antifouling properties of silicones, the PDMS-based coatings have a limited practical use because they are mechanically weak and easily damaged. Some of the authors have reported that the incorporation of very low amounts (0.5% *w*/*w*) of multi-walled carbon nanotubes (MWCNTs) into the silicone matrix spectacularly improves the physical and rheological behavior of the PDMS resin [10]. This was found to trigger significant changes in the nanocomposite surface topography, concomitant with variations in the wettability behavior upon immersion in water. This restructuring of the surface allows for an enhancing of the fouling-release performance of the coatings towards two major fouling species, the green macroalga (seaweed), Ulva linza, and the barnacle, Balanus amphitrite [11]. The obtained results suggest that, independently of the bulk mechanical performances, the surface properties significantly affect the fouling-release behavior of the filled materials.

A great variety of methods have been reported to increase surface roughness, including UV reactions [12], UV grafting [13,14], UV/ozone reactions [15,16], laser/plasma etching [17,18], chemical surface treatments [19], sequential growth of polymer layers [20,21,22], surface casting [7], and nanofiller loading [23,24,25,26]. All mentioned procedures present relative advantages and drawbacks. For instance, UV/ozone methods currently require two spectral lines, the first to produce ozone and the other to generate oxygen from ozone; oxygen plasma methods introduce high-energy ions and radicals, which render several coupled oxidations; UV grafting currently requires the adsorption of a photo-initiator; etc. [27]. Therefore, the search for simple new methods to increase the surface roughness of polymers continues. 

We note that the wide variety of methods can be classified in two large groups. One group contains those methods for which the roughness is increased because the polymer structure is chemically modified. These methods are highly reactive towards the polymer matrix, typically due to using UV reactions, as mentioned previously. The other group is formed by those methods that do not produce a chemical attack of the polymer—mainly those based on incorporating nanofillers in the matrix. For instance, it has been reported that the fine random dispersion of MWCNTs in PDMS, forming PDMS/MWCNT composites, increases the surface roughness [28]. An interesting alternative not explored in the literature as a method of inducing roughness, as far as we know, is to prepare structured composites for which nanofillers are preferentially oriented in a direction perpendicular to the surface. Our group has extensive experience with preparing structured composites using magnetic nanofillers, such as cobalt ferrites (CoFe_2_O_4_), which are oriented by magnetic fields applied during curing or solvent evaporation [29,30,31,32,33,34,35,36,37,38]. These works suggest the possibility of increasing the roughness by preparing composites containing not only MWCNT, but also CoFe_2_O_4_ magnetic nanoparticles, and curing the composite in the presence of a magnetic field perpendicular to the surface, generating structured PDMS/MWCNT/CoFe_2_O_4_ composites. 

Therefore, the aim of the present work is to explore the possibilities of inducing a roughness increase in PDMS films by two approaches, each associated to a mentioned group of methods: (i) structured composite (PDMS/MWCNT/CoFe_2_O_4_) curing in the presence of a magnetic field perpendicular to the surface; and (ii) surface attack through bromide radicals generated by UV reactions. The surface modifications induced using both methods were analyzed and then compared. 

## 2. Materials and Methods

### 2.1. Chemicals

PDMS Sylgard 184 (from Dow Corning, Midland, MI, USA) was used as a silicone elastomer consisting of two parts, A and B, which needed to be mixed together in a ratio of 10:1 in order to obtain the cross-linked material via a hydrosilylation reaction. According to the supplier, the formulation contained an α,ω-vinyl-terminated poly(dimethylsiloxane) polymer (*DP* = 434), a poly(dimethylsiloxane-*co*-hydrogenomethylsiloxan) copolymer, a vinyl resin additive and a platinum-based catalyst.

Bromine (Br_2_) blisters were obtained from Merck, Kenilworth, NJ, USA. MWCNTs were provided by Sigma Aldrich (St. Louis, MI, USA Code 6941855G; average length: 0.5–200 μm; average diameter: 7–15 nm). Na_2_S_2_O_3_ was provided by Anedra (Buenos Aires, Argentina). All solvents and reagents were of analytical quality and were used as received.

The synthesis and characterization of cobalt ferrites (CoFe_2_O_4_; 15 nm average diameter) of a high purity and crystallinity were reported in previous works [30,38]. 

Cover-slips for optical microscopy were used as glass substrates for preparing the films. The cover-slips were cleaned beforehand with acetone, ethanol and distilled water. A few films were prepared on an aluminium substrate (Sigma-Aldrich, St. Louis, MO, USA) under a similar cleaning procedure.

### 2.2. Preparation of PDMS/Filler Composite Films

PDMS base and cross-linker agents were mixed in proportions of 10:1 (*w*/*w*) at room temperature. Typically, 2 g of the base/cross-linker agent was used. Then, toluene was added (1 mL) to aid mixing and to ensure faster air bubble removal. This mixture was left under magnetic stirring until most of the toluene had evaporated, to obtain a viscous fluid system with an adequate fluidity for preparing the films. Composites with nanofillers dispersed in PDMS were also prepared using MWCNTs, by adding different amounts of MWCNTs to a solution of PDMS in toluene, before curing. The nominal weight fraction of MWCNT was about 1% *w*/*w* (without considering the toluene). 

To prepare the films, the viscous suspension was deposited onto glass substrates by spin-coating at room temperature (SPIN-1200D MIDAS SYSTEM spin-coater, Daejeon, Korea); 10 s at 2000 rpm followed by 15 s at 4000 rpm). Then, the samples were placed into an oven at 110 °C to evaporate the rest of the toluene and to cure the polymer (cross-linking process). 

Some samples were prepared using a different method. On the glass substrate, two adhesive tapes were glued, separated by a distance of 2–3 cm. In the region between the two tapes, the viscous suspension of PDMS/fillers was poured and then slowly spread using a spatula or a plastic ruler. This system was then cured thermally. In this way, films of the composite deposited on the substrate were obtained, with thicknesses close to that of the adhesive tapes (thickness of the tape: 130 μm). This method is referred to here as the tape method.

As mentioned previously, a few samples were prepared via the two methods (spin-coating and tape methods) on an aluminium substrate, for comparison, following the described protocols.

In some cases, CoFe_2_O_4_ particles were also added simultaneously with the nanotubes (in these cases: MWCNT ≈ 1% *w*/*w*; CoFe_2_O_4_ ≈ 2% *w*/*w*). Some of the samples containing CoFe_2_O_4_ were cured at room temperature under the application of a uniform magnetic field in a direction perpendicular to the surface. To perform this, the films formed on the substrate were placed between two rare-earth permanent magnets (samarium-cobalt alloys; disk shaped; flat surfaces; 36 mm diameter) immediately after the spin-coating process. The system was left between the magnets at room temperature until the toluene had completely evaporated and the polymer had cured. The magnetic field between the two magnets was close to the surface of the films and was, at its center, about 0.36 T (measured with a Hall probe sensor, Allegro Probe Model 1302A, Worcester, MA, USA). 

At least four replicates were prepared for each of the systems; that is, every time a specific experimental condition was changed (substrate, method of preparation, composition, etc.), at least four replicates were prepared. 

### 2.3. Exposition to Br_2_ Vapours and UV Reaction

**Figure 1:**
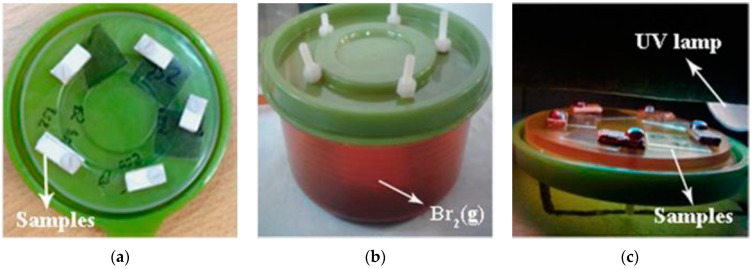
(**a**) Internal view of the cover in which sample holders were placed. (**b**) Container with liquid bromine in equilibrium with its vapour. The samples were located upside down on the lid; thus, the films were in contact with bromine vapours. (**c**) The lid was removed from the vessel and samples were placed face-up and UV-irradiated.

The set-up used in order to expose PDMS/MWCNT composite films to Br_2_ vapours, to be followed by UV irradiation, is shown in Figure 1. Briefly, the samples prepared by the methods described in the previous section, without removal from their substrate, were placed faced down into a 175 mL hermetic vessel. Immediately before closing the vessel, 0.5 mL of liquid bromine was introduced into its bottom using a Pasteur pipette. Once the vessel was closed, the bromine did not evaporate completely, but a liquid–vapour equilibrium was established inside the chamber. Thus, the Br_2_ (gas) pressure was estimated to be about 0.2–0.3 bar, which is the estimated vapour pressure of bromine at 25 °C [39]. The films were in contact with these vapours for 30 min (the presence of reddish Br_2_ vapours can be observed in Figure 1b). Afterwards, the films were removed from the vessel; some of the films were exposed to UV radiation from a medium-pressure Hg-lamp (5 W) placed 2 cm from the samples (Figure 1c) during a variable exposure time (30 min or 1, 2, 3 or 4 h). Then, the samples were washed with Na_2_S_2_O_3_ and water and placed in an oven for 30 min at 70 °C to remove possible drops of Br_2_ (liquid) condensed on the films.

### 2.4. Instrumentation

The thicknesses of the dried films were measured using a surface profilometer (Veeco, model Dektak 150, Plainview, NY, USA), whose instrumental details are described in a previous work [36]. The thickness of the film, *L*, was measured as a function of the scanned distance, and the average values of *L*, referred to as *<L>*, were calculated within a defined scanning distance range (4000 μm, depending on the sample) starting from at least 100 μm from the edge of the film.

Fourier transform infrared (FTIR) spectra (4000–400 cm^−1^; resolution 4 cm^−1^) were acquired with Nicolet 8700 (Madison, WI, USA) equipment using a Smart Orbit ATR accessory (single horizontal reflection with a diamond crystal) and a DTGS detector.

The structure of the dried composite films was investigated by scanning electron microscopy (SEM) using a field emission scanning electron microscope (FESEM; Zeiss Supra 40 Gemini, Oberkochen, Germany).

Atomic force microscopy (AFM) and magnetic force microscopy (MFM) [40] images were acquired to characterize the surfaces’ topography and the magnetic surface properties of the composites. A Bruker Multimode 8 SPM (Santa Barbara, CA, USA) and NanoScope V Controller (Billerica, Santa Barbara, CA, USA) were used. The image analyses were performed using Gwyddion version 2.46 (Brno, Czech Republic) and Nanoscope version 9.1 software (Santa Barbara, CA, USA). The AFM images were acquired in the intermittent mode using silicon tips with a spring constant of 1–5 Nm^−1^ and a resonance frequency in the range of 60–100 kHz. Areas of typically 50 μm × 50 μm were scanned. The MFM images were obtained in the lift mode, while tapping was used to record the magnetic signal using the phase-detection mode. Magnetic probes (Co/Cr; model MESP) provided by Bruker were employed to acquire the MFM images. The tips were magnetized before use. The lift height was set close to 100 nm, and the scan size was 10 μm × 10 μm.

For each AFM (or MFM) image, a reference plane (mean plane) was defined, and a *Z*-axis, perpendicular to that plane, was considered, where *Z* = 0 was on the plane. *Z*-values were calculated from the images in a discrete manner, where *Z_j_* was defined as the height of the *j*th-pixel from the mean plane. That is, *Z* is a discrete stochastic variable. Positive *Z*-values are associated to protrusions above the mean plane, while negative *Z*-values, to depressions below the plane. The average surface roughness (*R*_a_) of each AFM image was determined as the average deviation of height values from the mean plane, when considering *N* pixels in a given image: $R_{a}=\frac{1}{N}\sum_{j=1}^{N}\left|Z_{j}\right|$ (*N* = 262,144). For each sample, three AFM images (taken at different regions on the sample surface) were recorded. Considering that 4 replicated samples were prepared and analyzed, then the reported *R*_a_ values for each type of sample consisted of an average value and a standard deviation over 12 determinations.

Other related roughness stochastic variables are usually considered, such as the “well depth” and heights profile. The well depth is the maximum variable value calculated as |*Z_j_*| when considering only the negative *Z_j_*, that is, those associated to each well (below the mean plane). Then the mean well depth and the associated standard deviation can be calculated, analogously to the calculation of *R*_a_ (but *N*, in this case, is equal to the number of wells). 

Finally, when the height values *Z_j_* are taken on a defined (arbitrary) line in the plane, they are usually referred to as height profiles on a line, and are plotted against the position on the defined line.

## 3. Results and Discussion

No influence of the substrate used for depositing the films (glass and aluminium) and for the preparation method (spin-coating and tape methods) was observed. The results described here were independent of the substrate and preparation method. 

### 3.1. Surface Roughness in PDMS/Filler Composites

**Figure 2:**
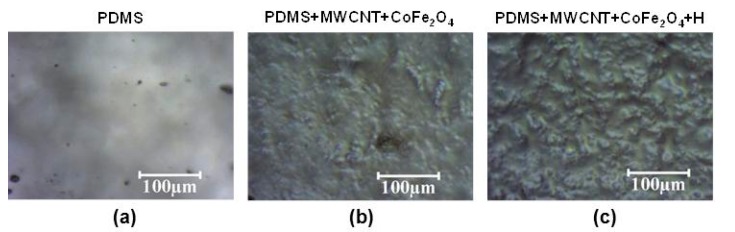
Optical microscopies: (**a**) PDMS. (**b**) PDMS + MWCNT (1%) + Fe_2_CoO_4_ (2%). (**c**) PDMS + MWCNT (1%) + Fe_2_CoO_4_ (2%) + **H**.

**Figure 3:**
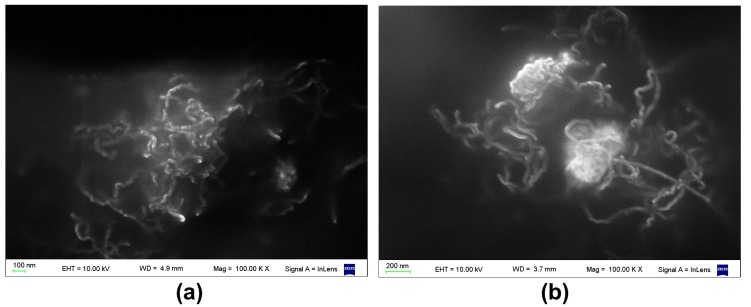
SEM images: (**a**) PDMS/MWCNT (MWCNT: 1% *w*/*w*). (**b**) PDMS/MWCNT/CoFe_2_O_4_ (MWCNT: 1% *w*/*w*; CoFe_2_O_4_: 2% *w*/*w*). The bright areas correspond to signals originating from the heaviest elements associated to CoFe_2_O_4_ agglomerates.

The thickness of all samples prepared by spin-coating was in the range of 20–40 μm, and was typically 30 μm. The samples prepared by the tape method (described in Section 2.2) had thicknesses of about 150 μm, which was close to the thickness of the tape. The presence of MWCNTs was detected in PDMS/MWCNT films by optical microscopy (Figure 2) and SEM (Figure 3). Moreover, the changes in surface features are clearly illustrated in the photographs of Figure 2 (taken with an optical microscope). It can be seen (qualitatively) in Figure 2 that the roughness increased when progressing in the series from PDMS to PDMS/MWCNT/CoFe_2_O_4_ to PDMS/MWCNT/CoFe_2_O_4_ + **H** (cured in the presence of the magnetic field **H**).

In order to obtain SEM images with an acceptable resolution, as for those observed in Figure 3, a 10 kV electron source was used instead of the common 3 kV source (that is, electrons with higher energy were used). This suggested that the nanomaterials (MWCNT and CoFe_2_O_4_) were covered by a layer of PDMS, thus requiring more penetrative electrons. 

The SEM images of MWCNTs dispersed in PDMS (Figure 3) were representative of the whole surface. On the other hand, the SEM images of samples that were cut under liquid nitrogen in the direction perpendicular to the surface indicated that there were MWCNTs close to the surface, which were detected at a depth of no greater than 5 μm from the edge. 

**Figure 4:**
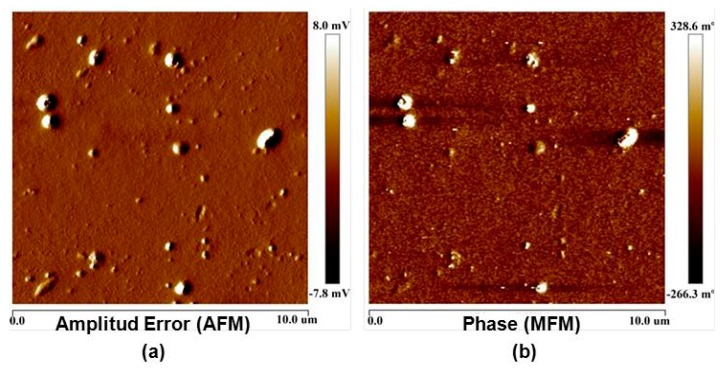
PDMS/MWCNT/CoFe_2_O_4_ cured in the presence of **H** (MWCNT: 1% *w*/*w*; CoFe_2_O_4_: 2% *w*/*w*). (**a**) AFM image. (**b**) MFM image. Exactly the same region of a given sample was scanned using the two techniques. The coincidence between both images suggests that the magnetic and non-magnetic materials were grouped to form clusters, as also observed by SEM in Figure 3.

Additionally, as can be seen in Figure 3, for PDMS/MWCNT/CoFe_2_O_4_ composites, it was always observed that CoFe_2_O_4_ particles were grouped in clusters of 300–600 nm in size, which were surrounded by MWCNTs (Figure 3). This result was confirmed by simultaneously recording AFM and MFM images (Figure 4), and observing that magnetic signals recorded by MFM (provided by the magnetic particles) were spatially coincident with the AFM signals (provided by both MWCNTs and magnetic particles), indicating that the nanotubes and magnetic particles were grouped, forming MWCNT/CoFe_2_O_4_ clusters. That is, no “free” magnetic nanoparticles (not grouped to a MWCNT) were detected, either by SEM or by AFM.

We made quantitative determinations of the roughness, given by the arithmetic average of the heights defined in Section 2.4 (Instrumentation) through the parameter *R*_a_. Other criteria, such as geometric averages or RMS values, are equivalent, and, in fact, are rendered to roughness parameters that are proportional to *R*_a_, in the sense that when *R*_a_ increases, they increase also. 

**Figure 5:**
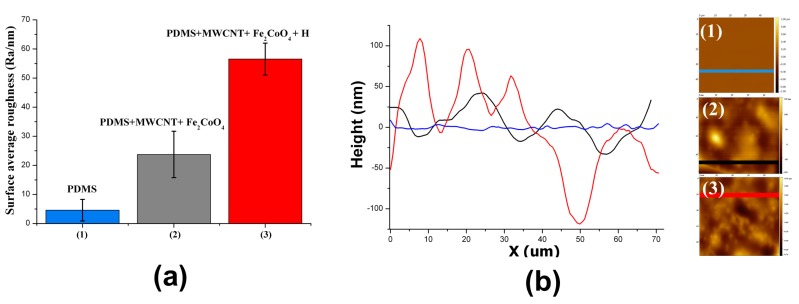
PDMS/filler composites: (**a**) Surface roughness. The altitude of the bars is the average roughness, *R*_a_, considering four replicates for each kind of material and three images for each sample. That is, each reported *R*_a_ was calculated using 12 AFM images. The respective standard deviation is indicated on each bar. (**b**) Height profiles recorded on the straight lines indicated in the insets. (1) PDMS, (2) PDMS/MWCNT/CoFe_2_O_4_, and (3) PDMS/MWCNT/CoFe_2_O_4_ cured in the presence of **H**. MWCNT: 1% *w*/*w*; CoFe_2_O_4_: 2% *w*/*w*.

The dispersion of nanoparticles (PDMS + CoFe_2_O_4_, without adding MWCNTs) did not introduce significant changes in the roughness. In contrast, the roughness increased by a factor of 3–5 in PDMS + MWCNT composites and in PDMS + MWCNT + CoFe_2_O_4_ (the factor is considered with respect to PDMS in both cases). An additional increase in the roughness was observed in the systems cured in the presence of the magnetic field (**H**); the roughness in PDMS + MWCNT + CoFe_2_O_4_ + **H** was between 2 and 3 times greater than in PDMS + MWCNT + CoFe_2_O_4_. This increase in the surface roughness parameter *R*_a_ is clearly noted in the results presented in Figure 5. 

The above results demonstrate that curing the magnetic composite under the action of a magnetic field induces a substantial increase in the surface roughness. The increase is expected to be dependent on the proportion of MWCNTs and magnetic nanoparticles dispersed in the polymer, and the intensity of the magnetic field. Although a systematic study of these variables is beyond the scope of the present work, the results are very conclusive concerning the effect of an enhanced roughness by applying magnetic fields in PDMS/MWCNT/CoFe_2_O_4_ composites.

### 3.2. Films Exposed to Br_2_ (Liquid), Br_2_ (Gas) and UV

In a first series of experiments, the films were impregnated with Br_2_ (liquid). Liquid drops of Br_2_ were deposited on the surface of the films by using Pasteur pipettes, under hood. The liquid mostly evaporated, although a part remained adsorbed (as the reddish colour, typical of bromine, was still observed on the films). After irradiating these samples with UV light for 1 h, it was observed with the naked eye that fractures and interruptions of the films were induced. The appearance of lines (scratches) in the films—randomly oriented, with dimensions in the order of 5 mm long and 1 mm wide, and corresponding to regions where the polymer film appeared destroyed—was detected, leaving the glass substrate exposed. That is, the mentioned treatment produced macroscopic damages, which could be observed by the naked eye. These damages were not detected in non-irradiated samples. 

Therefore, considering that liquid bromine impregnation produces (after UV irradiation) ruptures of the films at the macroscopic level, it was then decided that the samples would be exposed to bromine vapours. For this purpose, in a second series of experiments, the set-up and procedure described in Section 2.3 and Figure 1 were used in order to expose the films not to Br_2_ (liquid), but to Br_2_ (gas). After irradiation with UV light of the samples exposed to Br_2_ vapours, no damage, such as that observed when impregnating with liquid bromine, was observed under an optical microscope. However, the results of ATR, SEM and AFM showed significant changes in the samples at the microscopic level, as detailed below.

In the following, we refer exclusively to samples that were exposed to bromine vapours and then irradiated. The presence of bromine was detected by energy-dispersive spectroscopy (EDS; coupled to the SEM instrument) in samples that were exposed to Br_2_ (g), but not irradiated. Moreover, in those samples exposed to Br_2_ (g) that were not washed with a saturated solution of Na_2_S_2_O_3_ before UV irradiation, the presence of micro-spherical drops of 200 nm diameters, which were attributed to adsorbed bromine agglomerates on the surface, was detected by SEM. These micro-droplets were not present in the samples that were washed with thiosulfate.

**Figure 6:**
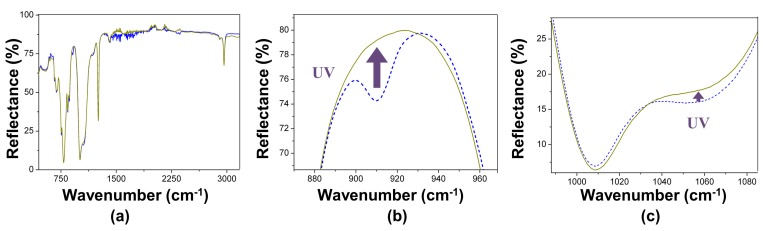
FTIR-ATR spectra of PDMS films exposed to Br_2_ vapours before (…) and after (-) UV irradiation: (**a**–**c**) correspond to zooms of different spectral regions.

The FTIR spectra of all irradiated (and washed) samples are shown in Figure 6. The spectra of the different samples presented similar characteristics, regardless of the substrate (aluminium or glass) and the method of preparation (spin-coating or tape methods). In some samples prepared on the glass substrate, the obtained spectrum was mounted on a very wide band of between 200 and 1000 cm^−1^, which is assigned to glass; in some of the samples prepared on aluminium by spin-coating, it was observed that the bromine attacked the substrate (before UV irradiation).

Figure 6b shows the irreversible disappearance, after UV irradiation, of the band at 910 cm^−1^, which corresponds to the double bonds, –C=CH_2_, of vinyl terminals of the siloxane chains [36]. It was also systematically observed that, after irradiation, there was a (partial) disappearance of the shoulder at 1060 cm^−1^ (Figure 6c), which is associated to Si–O–Si bonds [41]. No signals associated to MWCNTs (currently at 3400 and 1550 cm^−1^ [42]) were detected in PDMS/MWCNT composite films, likely due to a lack of instrumental sensitivity for the MWCNT concentrations used here. In summary, the FTIR spectra show that UV irradiation of the samples previously exposed to bromine vapours caused irreversible ruptures of the polymer chemical structure.

The following is a reasonable scheme of the mechanism of radical attack by Br• radicals generated by UV radiation:


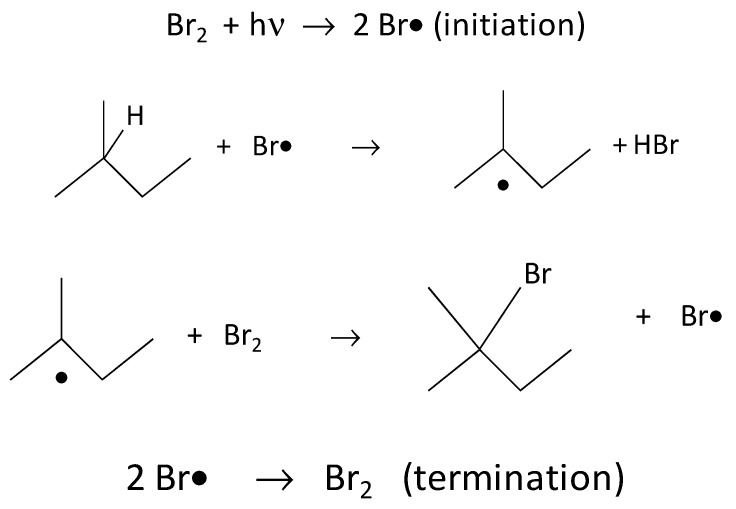


**Figure 7:**
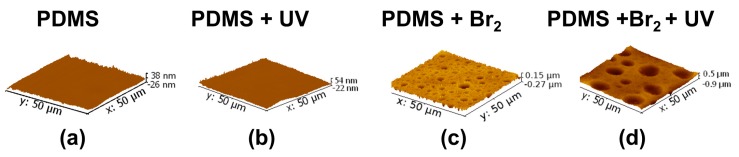
Examples of 3D AFM images: (**a**) Pristine PDMS. (**b**) UV-irradiated sample. (**c**) Sample exposed to Br_2_ (g), but not irradiated. (**d**) Sample exposed to Br_2_ (g) and then UV-irradiated.

**Figure 8:**
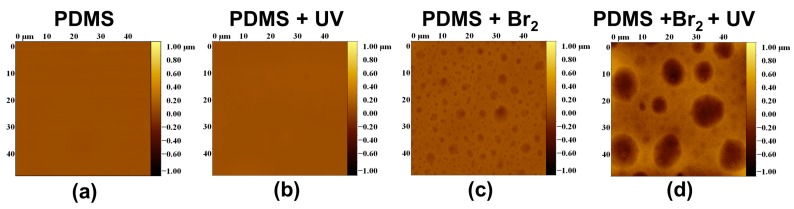
Examples of 2D AFM profiles: (**a**) Sample of pristine PDMS. (**b**) UV-irradiated sample. (**c**) Sample exposed to Br_2_ (g), but not irradiated. (**d**) Sample exposed to Br_2_ (g) followed by UV-irradiation.

A large change in surface characteristics was observed in the samples exposed to bromine vapours. Figure 7 and Figure 8 present 2D and 3D AFM images, respectively. The samples that were not exposed to bromine vapours appeared “flat”, compared to those that were. 

**Figure 9:**
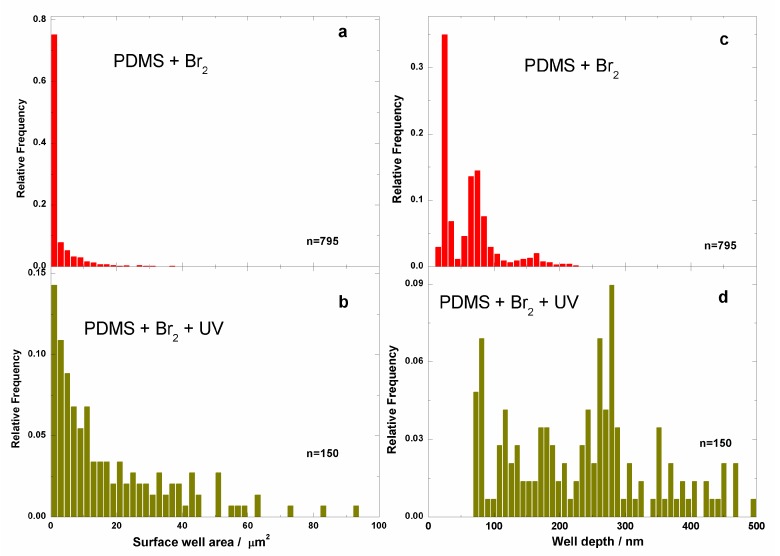
Well surface area histograms for bromine-treated samples: (**a**) PDMS + Br_2_ composite, and (**b**) PDMS + Br_2_ + UV composite. The well depth histogram for bromide-treated sample: (**c**) PDMS + Br_2_ composite, and (**d**) PDMS + Br_2_ + UV. Here, *n* is the number of wells detected in the 12 AFM images used to produce the histograms.

It is clear that exposure to bromine vapours in the absence of UV irradiation with the lamp, likely due to spurious UV from the ambient source, induced the appearance of surface wells, presenting the given distribution of well areas and depths: areas from 0 to 30 μm^2^ and depths from 15 to 225 nm (Figure 9a–c). After irradiation with the UV lamp, the distribution of surface areas and depths became broader, and wells with surface areas from 0 to 95 μm^2^ and a depth distribution from 75 to 500 nm were observed (Figure 9b–d). 

**Figure 10:**
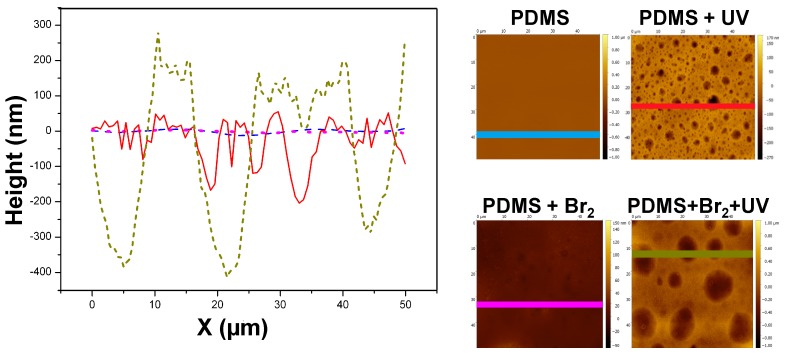
Height profiles of AFM, calculated on the images and lines shown below. The symbol X (μm) represents a distance on the indicated straight line, measured from the **left** to the **right**.

Figure 10 shows the height profiles (in nm) calculated on a straight line of 50 μm length for each sample. Clearly, the height profiles follow the same trend as *R*_a_: PDMS ≈ PDMS + UV < PDMS + Br_2_ < PDMS + Br_2_ + UV.

**Figure 11:**
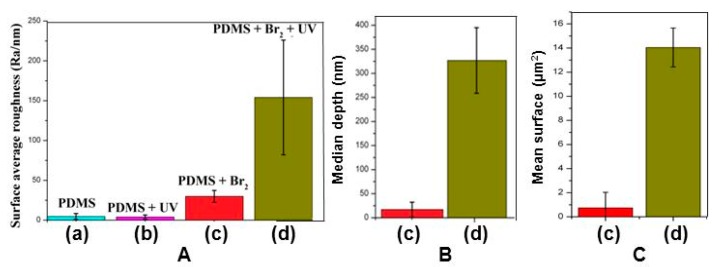
(**A**) Average surface roughness, *R*_a_. (**B**) Mean well depth for the bromide-treated samples. (**C**) Mean surface area of wells. The dispersions of four replicated samples (considering three images per sample) are indicated by segments on each bar.

The roughness observed by AFM increased in the expected sequence: PDMS ≈ PDMS + UV < PDMS + Br_2_ < PDMS + Br_2_ + UV. The mean roughness *R*_a_ and standard deviation (average values on four replicates for each composition) are shown in Figure 11A. The mean depths for wells presented in samples treated with bromide are shown in Figure 11B. 

## 4. Conclusions

The two methods explored in this work led to an increase in the surface roughness, which was quantified by the parameter *R*_a_. In the first, the use of a more physical method, which used MWCNT/CoFe_2_O_4_ fillers, gave an increase in the roughness when the PDMS films were cured in the presence of a magnetic field, **H**, which was remarkable in comparison to those cured without the presence of **H**. For example, in systems that were not cured with **H**, *R*_a_ increased by a factor of 5 when nanotubes and nanoparticles were dispersed, while the increase was 11-fold when these systems were cured in the presence of **H** perpendicular to the surface.

On the other hand, the chemical method of exposure to bromine vapours followed by UV irradiation produced drastic surface changes; wells had depths in the range from 75 to 500 nm. Although the wells were generated by the exposure to bromine vapours (with the possible influence of spurious UV radiation from the ambient source), the area of these wells increased by a factor of 3 when irradiating with the UV lamp. 

Although the primary aim was not to compare the methods, the question addressed to the comparison arises naturally. For instance, the physical method did not generate large surface wells, which were induced on the surface by the chemical attack, but many properties of the films changed when filler particles were added. For example, the nanocomposites displayed superparamagnetism when small magnetic nanoparticles were included, and in some cases, the films may have become electrical conductors if relatively large amounts of MWCNTs were dispersed. These changes in physical properties induced by the physical processes may or may not be of relevance for applications. For instance, in the case of applications as antifouling agents, the mentioned changes are not of central importance; the relevant variable is the change of surface topology, given by the increase of roughness, the creation of wells, etc. On the other hand, in the case of applications for microfluidics, the generation of magnetic surfaces can be of central importance for designing micro-valves or actuators.

In the physical method, the roughness could be modified mainly by changing the amount of MWCNTs. We verified that the roughness increased when increasing the proportion MWCNT/PDMS. From a practical point of view, the amount of MWCNTs is limited by costs and by the fact that the above concentration threshold in the presence of MWCNTs can drastically perturb many physical properties of the composite, such as electric and thermal conductivities.

In the chemical method, the exposure to UV radiation was the main factor to control (the concentration of Br_2_ adsorbed on the surface is hard to change, as it is determined by the bromine vapour pressure at room temperature). If the incident power is fixed, then the roughness is increased by increasing the exposition time, up to a plateau. For instance, we have observed that, under the present instrumental conditions, no further roughness increase was noted for exposition times above 15 minutes. Below this time, it is possible, with the experimental set-up used in this work, to have a (partial) control of the roughness by changing the exposition time, although a series of systematic studies are required to quantify the effects on the roughness.

A comparison of the effect on the roughness between both methods is possible; however, it requires some care, as it is clear that the chemical method does generate wells, while the physical method does not. Nevertheless, we can say that the largest roughness increase was obtained for (PDMS + Br_2_ (gas) + UV). Moreover, defining the relative roughness *R* as $R\equiv \frac{R_{a}-R_{a}(\mathrm{PDMS})}{R_{a}(\mathrm{PDMS})}\times 100$, then the following sequence is obtained: *R*(PDMS) ≈ *R*(PDMS + UV) ≈ 0 < *R*(PDMS + MWCNT + CoFe_2_O_4_) ≈ *R*(PDMS + Br_2_ (g)) ≈ 400 < *R*(PDMS + MWCNT + CoFe_2_O_4_ + **H**) ≈ 1000 < *R*(PDMS + Br_2_ (g) + UV) ≈ 3000. Although these values are dependent on the concentration of nanomaterials, the intensity of the magnetic field, and the UV conditions, they clearly indicate that both—(nanomaterials + **H)** and (Br_2_ + UV)—induce large surface changes.

The increase of *R*_a_ reported in the present work, for systems that were not cured in the presence of magnetic field, is similar to those reported by other authors [10,11,26], although our values and the values reported by these authors were both dependent on the specific conditions. However, it is noteworthy that curing in the presence of magnetic fields induces *R*_a_ values greater than those previously reported. On the other hand, the chemical attack method induced by bromine radicals generated by UV radiation produces not only an increase in *R*_a_, but also the occurrence of wells, whose depths are quantified by the mean height parameter. Although, to the extent of our knowledge, we have not detected reports of a quantification of these effects in the literature, the *R*_a_ parameter reported here for the chemical attack process was even greater than that measured for the physical process. The surface effects were, in this case, similar to or higher than those reported in references [12,18,19].

Both processes reported here are suitable for different applications. For instance, the physical process using the nanocomposite approach is interesting for cases in which the nanomaterial is not only responsible for the surface roughness increasing, but also poses a well-defined physical property, such as electrical conduction, thermal conduction, magnetism or magneto resistance. Considering the case of developing micro-valves for biochemical applications in which the surface of the materials is modified by using magnetic fillers, the roughness increase may avoid/reduce the formation of bacterial films, while the magnetic character can be used to open/close a valve. In the case of developing sensors, the roughness increase can provide adsorption sites for an analyte, while electrical signals are driven through the composite if conducting nanofillers are included. Thus, although the physical approach is relatively expensive, as it requires loading the polymer with nanomaterials, it finds applications in sensors and actuators for which a modified surface is required in combination with a response given by a physical property (magnetism, conductivity, etc.). On the other hand, the chemical attack does not require nanomaterials; thus, the costs are, in principle, lower, in comparison to the nanocomposite approach. The chemical process does not introduce a new physical property to the polymer (such as magnetism), it simply creates strong perturbations to the topology of the films. Thus, the chemical attack seems to be more suitable for situations in which it is needed only to protect a surface for biofilm formation.

To the extent of our knowledge, the present study constitutes the first report of increasing surface roughnesses using the described methods. Each method induces a different characteristic on the surface, and the eventual selection of which is used will depend on the particular application desired.
